# Lost in transition? Loss of follow-up and quality of life in adults after resection of choledochal malformation in childhood

**DOI:** 10.1515/iss-2023-0061

**Published:** 2024-06-14

**Authors:** Reem Abo-Namous, Joachim F. Kuebler, Andrej Potthoff, Omid Madadi-Sanjani, Marie Uecker, Jens Dingemann, Claus Petersen, Benno Ure, Nagoud Schukfeh

**Affiliations:** Department of Pediatric Surgery, Hannover Medical School, Hannover, Germany; Department of Gastroenterology, Hannover Medical School, Hannover, Germany

**Keywords:** health-related quality of life, choledochal malformation, pediatric surgery, transition, hepatobiliary surgery

## Abstract

**Objectives:**

Choledochal malformation (CM) is a rare disease that can lead to malignancy and potential long-term sequelae despite surgical resection. There is no long-term follow-up data on patients after CM resection in Germany. We aimed to determine the long-term outcome of our patients with a duration of follow-up >10 years and focused on long-term sequelae and health-related quality of life (HRQOL).

**Methods:**

All patients who had undergone CM-resection in our department from 01/1978 to 06/2009 were contacted. Patients were interviewed about postoperative complications and their present medical attendance. HRQOL was determined using Pediatric Quality of Life Inventory 4.0 (PedsQL), version for adults. The PedsQL scales the HRQOL from 0 to 100, with higher scores indicating a better HRQOL. Scores were compared to those published for a healthy population.

**Results:**

Out of 56 patients who were contacted, 23 (41 %) participated. The median age at time of surgery was 3.1 years (6 days–16.1 years) and at time of the survey 24.3 years (11.1–53.8 years). Eighteen patients (78 %) had ceased their gastroenterologic follow-up at a median time of 4.3 years after surgery. Five (22 %) were still in gastroenterologic follow-up, two of these had an uneventful clinical course, and three (13 %) had ongoing complications attributed to the CM. One of these had undergone hemihepatectomy 34 years postoperatively due to bile duct stenosis, one had undergone removal of bile duct stones 14 years postoperatively, and one suffered from portal vein thrombosis with esophageal and jejunal varices. There was no mortality in our series. Median total HRQOL score was 89. There was no significant difference in the median total health, physical health, and psychosocial health scores of our patients in comparison to the healthy population.

**Conclusions:**

We confirmed that the majority of patients after CM resection are lost to follow-up. Those who answered our questionnaire showed a good HRQOL. Given the high rate of severe long-term complications and the life-long risk of malignancy, we recommend a transition program for all patients.

## Introduction

Choledochal malformation (CM) is a rare disease with surgical resection mostly performed in infancy or during childhood [1, 2]. With 1:1,000 live births, its incidence in Asian countries [3], [4], [5] is considerably higher than in Europe, where actual numbers report on an incidence of 1:59,000 live births in the Netherlands [6]. The reason for this Asian predominance is not clear yet [3].

Patients with CM have an increased risk of hepatobiliary malignancies [7]. However, hepatobiliary tumors associated with CM usually occur during adulthood [8] and in rare cases in children or adolescents after CM resection [7]. Reports on malignancies following CM resection mostly were from Asian countries. In contrast, European follow-up studies from the United Kingdom and the Netherlands did not observe any malignancies following CM resection [6, 9]. Nevertheless, lifelong postoperative follow-up including sonography and CA19-9-determination has been recommended for all patients who underwent CM resection [7].

Although a major number of patients after CM resection in childhood have now reached adulthood, there are no studies reporting on the transition of patients after CM resection in to adult medical care. There is also no data on the quality of life of patients following CM resection. We hypothesized that the majority of patients who had undergone CM resection during childhood in Germany and now have reached adulthood are lost to follow-up and have never carried out transition into adult medical care.

## Patients and methods

The study was approved by the local ethics committee (Number 8230_BO_K_2018). We included all pediatric and adolescent patients who had undergone surgery for CM in our department from January 1978 to June 2009. Medical records of these patients were reviewed and data on operation and early postoperative course were analyzed retrospectively.

Additionally, all patients were contacted by post and offered an appointment in our outpatient clinic for follow-up. Patients who agreed to participate received the PedsQL questionnaire and a standardized questionnaire concerning their postoperative clinical course and their gastroenterologic follow-up investigations. Patients who did not respond to our letters were additionally contacted by telephone and asked about their clinical course including complications and their past and present gastroenterologic follow-up investigations using the above-mentioned standardized questionnaires. Contacting of the patients, telephone interviews and clinical examinations were carried out by two of the authors (R.A. and N.S.). Patients gave informed consent on their participation.

### Measurement of health-related quality of life

Health-related quality of life (HRQOL) was determined using a validated questionnaire (Pediatric Quality of Life Inventory 4.0 (PedsQL), version for adults). The 23-item Pediatric quality of life inventory (PedsQL) 4.0 Generic Core Scale encompasses physical functioning (8 items), emotional functioning (5 items), social functioning (5 items), and school functioning (5 items). These scales produce a total, psychosocial, and physical summary score [10]. Items are reverse-scored and linearly transformed to a scale of 0–100, with higher scores indicating better HRQOL. The evaluation tool is available in the German language and has undergone linguistic validation for academic use in Germany [The PedsQL™, Mapi Research Institute, Lyon, France]. A healthy cohort was derived from the previously conducted PedsQL 4.0 Generic Core Scales initial field test [11] and a statewide State Children Health Insurance Program evaluation from the United States [10]. The scores achieved by the patients in our present study were compared to those published for the healthy population described from children from the United States [11].

### Statistical analyses

Mean PedsQL scale and summary scores were calculated for all patients who signed the informed consent. Mean PedsQL scores were compared with those of a healthy population derived from the previously conducted PedsQL 4.0 Generic Core Scales initial field test [10] utilizing independent sample *t*-tests. Data are given in median and range unless stated otherwise.

## Results

### Patient cohort

Out of 56 patients (73 % female) who were contacted, 23 (41 %) agreed to participate. Median age at time of surgery was 3.1 years (6 days–16.1 years) and 24.3 years (11.1–53.8 years) at follow-up. Table 1 shows the characteristics of those patients who responded to our questionnaire in comparison to those who did not respond.

**Table 1:** Characteristics of responders versus nonresponders.

	Responders	Nonresponders
Number of patients	n=23	n=33
Female	87 %	64 %
Median age at surgery	3.1 years	2.9 years
Todani cyst type I	91 %	70 %
Open surgical approach	65 %	76 %
Short-term complications (<30 days)	17 %	27 %

Responders: patients who answered our questionnaire. Nonresponders: patients who did not answer our questionnaire.

Out of those patients who answered our questionnaire (responders), 21 (91 %) had a choledochal cyst type 1 according to the Todani classification [12], and two patients had a type 4 cyst.

### Surgical details

Patients had undergone resection of the extrahepatic biliary tree with restoration of the continuity via hepaticojejunostomy between December 1978 and June 2009. Until 2001, all were operated via open surgery until laparoscopic CM resection was introduced in 2002. In total, 35 patients underwent open operation, 21 patients were operated laparoscopically with seven conversions (33 %). Median operative time was 3:50 h (range 2:40–6:10 h) for laparoscopic and 4:25 h (range 1:55–6:10 h) for open surgery.

### Follow-up and short-term complications

Median time between surgery and our study was 19.9 years (range 10.3–40.9 years). Five out of 23 patients (22 %) were still in gastroenterologic follow-up at the time of our study (Table 1). All other patients had ceased their follow-up at a median of 4.3 years after surgery.

The overall complication rate was 26 %. Short-term complications included stenosis and revision of the hepatobiliary anastomosis after 6 months, torsion of the enteric anastomosis due to adhesions leading to re-laparotomy, and adhesiolysis at the seventh postoperative day and percutaneous bleeding requiring resuture at the first postoperative day in those patients who had ceased follow-up (Table 2).

**Table 2:** Surgical details, follow-up, and complications.

Number	Gender	Age at surgery, years	Age at study, years	OP type	In regular follow-up	Complications
1	f	1.1	31.7	Open	No	-
2	f	3.7	23.4	Open	No	-
3	f	13.4	48.9	Open	Yes	Bile duct stenosis^a^
4	f	0.0	25.2	Open	No	-
5	f	5.9	20.8	MIS	No	-
6	m	13.0	53.8	Open	Yes	Bile stones, recurrent cholangitis^a^
7	f	4.4	24.3	Open	No	-
8	m	0.5	11.1	Open	No	-
9	f	16.1	51.0	Open	No	-
10	f	1.3	39.6	Open	Yes	Portal vein thrombosis, liver fibrosis, esophageal varices^a^
11	f	12.4	40.9	Open	No	-
12	f	2.2	36.3	Open	No	-
13	f	1.7	13.4	MIS	No	-
14	f	3.1	16.7	Converted	Yes	-
15	f	7.1	17.4	MIS	No	-
16	f	4.2	18.3	Converted	No	Stenosis of hepatobiliary anastomosis
17	f	12.7	23.1	MIS	No	-
18	f	3.1	37.2	Open	No	-
19	f	0.2	17.7	MIS	No	Torsion of enteric anastomosis due to adhesions
20	f	0.1	15.3	Open	No	-
21	m	3.5	15.3	MIS	Yes	-
22	f	4.1	45.0	Open	No	Percutaneous bleeding
23	f	0.5	35.1	Open	No	-

^a^Long-term sequelae. Number: patient’s number in publication. OP type: open – conventional open CM resection; MIS, minimally invasive surgery (laparoscopic CM resection); converted – intraoperative conversion from laparoscopic to open CM resection. Complications: postoperative complications.

Two out of the five patients who were still in gastroenterologic follow-up both had undergone laparoscopic CM resection at the age of 3 years. One had been converted to open surgery. Both patients received yearly prophylactic examinations in spite of an uneventful postoperative clinical course during a follow-up period of 13 and 11 years, respectively.

### Long-term sequelae

Three out of the five patients who were still in gastroenterologic follow-up (60 %) had ongoing sequelae attributed to the CM (Table 2): One of these was a 48-year-old female who had received open resection of her CM at the age of 13 years 35 years prior to the study. Thirty-four years postoperatively, she developed a bile duct stenosis and a bile duct cyst in the right liver lobe. She underwent right-sided hemi-hepatectomy.

Another 53-year-old male patient had received open resection of his CM at the age of 13 years 41 years prior to our study. He developed bile stones that were removed endoscopically 14 years postoperatively. He had recurrent episodes of cholangitis in the further course.

A 39-year-old female had received open resection of her CM at the age of 14 months 38 years prior to our study. Postoperatively, she developed a portal vein thrombosis with consecutive liver fibrosis and esophageal varices with recurrent bleedings, splenomegaly, jejunal varices, and abdominal adhesions during the following 7 years. This patient received splenectomy, right-sided ovarectomy and had an abortion due to esophageal variceal bleeding. This patient had also ongoing recurrent episodes of cholangitis. All short-term complications and long-term sequelae concerned patients with Todani type 1 choledochal cysts. There was no influence of laparoscopic or open approach on the development of complications.

### Health-related quality of life (HRQOL)

**Figure 1:**
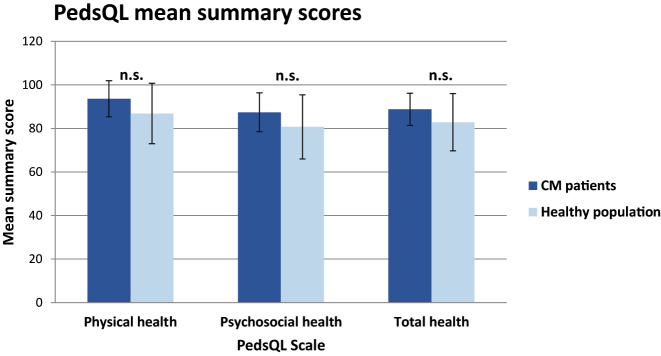
PedsQL mean summary scores. Mean summary scores in patients after CM resection compared with healthy population according to PedsQL [Varni et al. [11]]. Physical summary score consists of the PedsQL physical functioning score. Psychosocial summary score consists of emotional, social, and school functioning score. Total summary score consists of physical and psychosocial summary score. Data given in mean±standard deviation.

Median health-related quality of life scores were high for all patients: median total health score was 89; median physical health and median psychosocial health score were 94 and 87, respectively. There was no significant difference in the HRQOL scores of our patients in comparison to the healthy population (Figure 1). In detail, the mean physical functioning score was 94, the mean emotional functioning score was 80, the mean social functioning score was 96, and the mean school/work functioning score was 92.

## Discussion

Transition into adult medical care has become an important issue in the care of pediatric surgical conditions, especially hepatobiliary diseases [13, 14]. In the recent years, numerous studies dealt with the long-term outcome after CM resection, but these studies are focused rather on long-term complications than on patients’ transition into adult medical care [6, 15]. There is only few data from large cohorts of adult patients following CM resection in childhood.

In a recent publication from a single center in Japan [16], the authors evaluated the postoperative complications in 79 adult patients who had undergone pediatric CM resection. After a median postoperative period of 24.5 years, the authors found that 54.3 % attended postoperative follow-up. No patient had biliary carcinoma. Twelve patients (34.2 %) had postoperative complications. Out of these, six underwent regular checkups [16]. In another recent Japanese study, Mukai et al. [17] reported on the long-term outcomes of surgery for CM, focusing on follow-up and late complications. In their retrospective study including 110 patients, the authors found intrahepatic bile duct dilatation in 0.9 %, intrahepatic bile duct stones in 2.7 %, and adhesive ileus in 3.6 % of patients within 20 years after surgery. There was no biliary carcinoma in their series. Their overall follow-up rate was 37.3 %, and the follow-up rate declined to 14.5 % in patients who had undergone surgery more than 20 years previously to the study. The authors concluded that patients after CM resection should be followed-up by adult gastroenterologists [17].

In contrast, an increased risk of malignancy has been reported by numerous other Japanese authors [18], [19], [20]. In a large study from Japan including 1,353 pediatric and adult patients with choledochal malformation, the group reported an incidence of 16.2 % malignancy associated with primary CM and 0.7 % following CM resection [19]. Our group had conducted a review article and recommended a structured follow-up program following CM resection with yearly clinical, biomedical, and sonographic examinations of patients after CM resection [7]. In a recently published international survey among pediatric hepatobiliary surgeons and gastroenterologists, 91 % of respondents stated that a structured follow-up program after CM resection was available in their department [1].

The high drop-out rate from follow-up identified by Mukai et al. [17] is in accordance to our findings. We confirmed a drop-out rate of 78 % already at a median time of 4.3 years after CM resection. The majority of complications seen in our study were apparent in patients who fulfilled transition. Three patients had a complicated long-term postoperative course; in one patient, complications started after more than three decades of uneventful postoperative course.

Regarding the higher incidence of CM in Asian countries, data from Japan may not necessarily be transferable to European conditions. So far, there have only been two series dealing with long-term complications after CM resection in a European country. In a retrospective study [6], the short- and long-term complications in 91 pediatric patients who underwent CM resection in the Netherlands have been reported. No malignancy was identified during follow-up. The complication rate of 40 % (22 % short-term and 18 % long-term) was somehow similar to the rate of 35 % found in our study. Another European study has been recently published by Hyvärinen et al. who reported on the outcome after CM resection in 55 pediatric patients after a median follow-up of 5.8 years. In this collective, 21 % had long-term complications including one case of hepatocellular carcinoma [21]. According to the findings of Eijnden and in contrast to those of Hyvärinen [6, 21], we did not find any case of malignancy even in long-term follow-up in our collective. As recently published, we recommended life-long follow-up due to an elevated risk of malignancy [7]. Our present data confirm a loss follow-up in CM patients and emphasizes the importance of a life-long follow-up in CM patients in order to identify late postoperative sequelae in an early stage.

The only study on HRQOL in patients after pediatric CM resection until date was published by Baba et al. [16]. They compared the HRQOL of patients with postoperative vs. without postoperative complications. Among 79 patients, more than 18 years of age 35 (44.4 %) had answered the questionnaires after a median postoperative period of 24.5 years (range 5–31). HRQOL was significantly lower in patients with complications in all health domains. In our present study, we compared HRQOL of patients after CM resection to scores in a healthy population and found no significant difference between both groups. It may be speculated that this fact may substantially contribute to loss of follow-up. However, given the high rate of long-term sequelae we found in our cohort, the high drop-out rate might also be due to poor results and unwillingness to response.

Our present study has limitations. The first limitation is the low response rate, which limits the generalization of our findings. There might also be some bias in comparing the HRQOL of our German population with children from the United States. In addition, the small number of our patients makes statistical analysis difficult and impairs the explanatory power of our results. One weakness of both above mentioned studies of Mukai and Eijnden [6, 17] when compared to our present study was the sole retrospective aspect of using hospital databases to determine follow-up rate and complications. In contrast, we contacted patients and, therefore, were able to include also those ones who received follow-up investigations or medical treatment in other institutions. Furthermore, as most of our patients had a Todani type 1 cyst, and the two patients with a Todani type 4 cyst had no complications, we cannot make a statement whether the cyst type has an influence on the development of complications.

## Conclusions

We confirmed that the majority of patients after resection of a CM are lost to follow-up. We found no case of malignancy but identified some severe long-term sequelae following an initially uneventful postoperative course. Given the high rate of severe long-term complications and the life-long risk of malignancy reported from other authors, we recommend a transition program and systematic follow-up for patients after CM resection into adulthood.
